# Impact of caring for persons with Alzheimer’s disease or dementia on caregivers’ health outcomes: findings from a community based survey in Japan

**DOI:** 10.1186/s12877-016-0298-y

**Published:** 2016-06-10

**Authors:** Amir Goren, William Montgomery, Kristin Kahle-Wrobleski, Tomomi Nakamura, Kaname Ueda

**Affiliations:** Health Outcomes Practice, Kantar Health, 11 Madison Ave, Floor 12, New York, NY 10010 USA; Global Patient Outcomes & Real World Evidence, Eli Lilly Australia, 112 Wharf Rd, West, Ryde, NSW 2114 Australia; Global Patient Outcomes & Real World Evidence, Eli Lilly and Company, Lilly Corporate Center, Indianapolis, IN 46285 USA; Medical Development Unit, Eli Lilly Japan K.K, 7-1-5, Isogami-dori, chuou-ku, Kobe 651-0086 Japan; Health Outcomes, Health Technology Assessment, & Real World Evidence, Medical Development Unit, Eli Lilly Japan K.K, 7-1-5, Isogami-dori, chuou-ku, Kobe 651-0086 Japan

**Keywords:** Alzheimer’s disease, Dementia, Caregiving, Health outcomes, Japan

## Abstract

**Background:**

This study assessed how family caregivers for patients with Alzheimer’s disease (AD) or dementia in Japan differed from non-caregivers in characteristics and health outcomes (i.e., comorbidities, health-related quality of life [HRQoL], productivity, and resource use). Caregivers were hypothesized to experience significantly poorer outcomes than non-caregivers.

**Methods:**

Data were combined from the 2012 and 2013 National Health and Wellness Survey in Japan (*n* = 60000). Caregivers for adult relatives with AD or dementia were compared with non-caregivers on: comorbidities (including Patient Health Questionnaire (PHQ-9) cutoff scores suggesting presence/absence of major depressive disorder (MDD)), Work Productivity and Activity Impairment (WPAI), SF-36v2-based HRQoL, and healthcare resource utilization. Sociodemographic characteristics, health characteristics and behaviors, and Charlson comorbidity index (CCI) scores were compared across groups. Propensity matching, based on scores generated from a logistic regression predicting caregiving, was used to match caregivers with non-caregivers with similar likelihood of being caregivers. Bivariate comparisons across matched groups served to estimate outcomes differences due to caregiving.

**Results:**

Among 55060 respondents, compared with non-caregivers (*n* = 53758), caregivers (*n* = 1302) were older (52.6 vs. 47.5 years), more frequently female (53 % vs. 49 %), married/partnered, frequent alcohol drinkers, current smokers, exercisers, and not employed, and they averaged higher CCI scores (0.37 vs. 0.14), all *p* < 0.05. Propensity scores incorporated sex, age, body mass index (BMI), exercise, alcohol, smoking, marital status, CCI, insured status, education, employment, income, and children in household. A greedy matching algorithm produced 1297 exact matches, excluding 5 non-matched caregivers. Health utilities scores were significantly lower among caregivers (0.724) vs. non-caregivers (0.764), as were SF-36v2 Physical and Mental Component Summary scores. Caregivers vs. non-caregivers had significantly higher absenteeism, presenteeism-related impairment, overall work impairment (25.8 % vs. 20.4 %, respectively), and activity impairment (25.4 % vs. 21.8 %), more emergency room and traditional provider visits (7.70 vs. 5.35) in the past six months, and more frequent MDD (14 % vs. 9 %), depression, insomnia, anxiety, and pain.

**Conclusions:**

Those providing care for patients with AD or dementia in Japan experienced significantly poorer HRQoL and greater comorbid risk, productivity impairment, and resource use. These findings inform the need for greater support for caregivers and their patients.

## Background

Alzheimer’s disease (AD) is a progressive neurodegenerative disease posing a tremendous burden for patients, caregivers, and health care systems. Disease symptomatology includes, most notably, impaired cognition (e.g., memory difficulties), as well as impairments in daily activities and increasing functional dependence. This constellation of symptoms results in an increasing degree of care required, often provided by family caregivers [1]. The cause of AD remains incompletely understood and currently there is no cure. Currently available treatment is aimed at improving cognitive function and managing behavioral symptoms [2].

AD is the most common form of dementia, with dementia of the Alzheimer’s type (DAT) projected to increase dramatically over the coming decades due to the aging global population [3, 4]. Precise global prevalence estimates have been difficult to establish, but approximately 4.7 million older adults in the U.S. have been estimated to have AD [5]. In Japan in 2010, 2.5 million adults were estimated to suffer from dementia, ranking Japan among nine countries with the highest number of sufferers [6].

Studies have emerged to help better understand the challenges associated with an aging population in Japan. A nationwide survey in 2012 reported prevalence of dementia among Japanese aged 65 and over to be 15.8 %, with AD the most common form, reported among 67.6 % of patients in the same survey [7]. This rate was considerably higher than previous estimates of dementia in Japan that ranged from 2.4 %–8.5 %, with AD developed by 60.8 % of participants with dementia [8, 9]. Evidence suggests that rates of AD are increasing in Japan [10, 11] and that it represents a major health concern not only in Japan but across Asia [12]. The most recent estimates of prevalence suggest an AD rate of 3.8 % in Japan [10, 13].

Beginning in 2000, Japan has implemented an insurance program and approach to long-term care providing comprehensive evaluation of the elderly, as well as in-kind care and financial support for family caregivers [14]. In 2012, to provide further support for the aging population, the Japanese government delivered the Orange Plan for elderly people with dementia. This initiative includes action plans for early detection and early diagnosis, as well as funds to continue home care after the onset of dementia [15].

As noted, care demands are significant for those diagnosed with AD. The slow, progressive nature of this disease and functional impairment associated with symptoms result in many patients being cared for primarily by family members [16]. A growing body of research suggests that the progressive burden of care associated with AD can have real and significant deleterious health consequences for these family caregivers [17].

Empirical studies suggest that family caregivers across a number of disease conditions have a greater risk of medical illness, psychological impairment, disruption of professional and personal roles, and critically, an increased risk of mortality [18–20]. At the same time, evidence suggests that some caregivers find positive effects associated with caring for a family member diagnosed with a chronic or terminal disease. Indeed, across diseases such as cancer, heart failure, and AD, studies have documented benefit-finding and meaning associated with the caregiver role, and interventions have been developed that seek to reinforce and promote the positive effects of caregiving [21–24]. Cheng and colleagues have reported positive preliminary results of a benefit-finding intervention for AD caregivers in China, with reductions in depression reported by the intervention group [22].

Aside from negative and positive effects on mental and physical health, significant costs have also been recognized to be associated with informal AD care. In fact, a recent 2013 European study found that costs associated with informal caregiving compose the majority of total societal costs linked with AD (over 50 %) and increase even further with disease severity [25]. A recent survey revealed a similar situation in Japan, with the total 2014 societal cost for dementia estimated at $145 billion and the cost of informal care estimated at $62 billion (where $1 = 100 yen) [26]. A number of deleterious effects have been revealed about caregiver burden associated with AD [27], including physical [19, 28], psychological [29, 30], social [31], and financial [32] consequences, as well as similar findings beginning to emerge in developing countries [33].

Although a number of focused studies by Arai and others have documented substantial burden among family caregivers of the elderly in Japan, as well as the challenges associated with providing care, research quantifying the degree of burden experienced by AD caregivers across multiple outcomes (encompassing not just quality of life, but also productivity, comorbidities, and resource use) in the broader population is lacking [34–40]. In addition, a qualitative study in 2008 found that a number of factors were protective against caregiver burden, including a good prior relationship between caregiver and patient, and the utilization of resources by the caregiver [41]. The authors did note that certain dyads were at greater risk (e.g., husbands caring for wives), and they recommended services be put in place to support higher-risk circumstances.

In a recent study, Kamiya and colleagues investigated factors associated with caregiving burden and found that behavioral disturbances and lower scores on the Mini-Mental State Examination (MMSE) were associated with increased burden [42]. Finally, Hirono and colleagues examined the role of caregiver burden in the eventual institutionalization or death of patients with AD in Japan. The authors followed 150 individual dyads for at least one year and found that initial caregiver burden was a significant predictor of both outcomes: death and transition of care to an institution [43]. Critically, the authors found that the early use of resources to aid in care (namely, daycare and home care services) were once again protective factors that reduced burden. The authors concluded that caregiver education was needed to increase understanding of the meaning and necessity of supportive care services and their ability to reduce caregiver burden.

Interventions targeting caregivers have been the topic of emerging research, although little has been published in Japan. A 1999 study by Hosaka and Sugiyama investigated the effects of a pilot program for AD caregivers, reporting that a 5-week educational and supportive group program resulted in significant improvements in mood and functioning among caregivers [44].

Despite the lack of nationwide research on supportive care programs in Japan, in recognition of the high degree of burden experienced by AD caregivers, Hashimoto et al. reported the beneficial effect of donepezil hydrochloride (approved for treatment of DAT) on caregiver burden, finding that caregivers of symptomatic patients reported a significant decrease in burden over the course of the 12-week trial [45]. Hishikawa et al. reported that garantamine treatment of patients with AD reduced behavioral and psychological symptoms of dementia and corresponding caregiver burden in a six-month retrospective evaluation study [46], while Kuroda et al. reported that rivastigmine was also associated with reduced caregiver burden [47]. This, along with supportive therapies, represents an important but under-researched domain of investigation.

Large-scale population-based studies, particularly those that include both urban and rural participants, and a broad range of psychosocial and economic outcomes, provide critical information that can enhance our understanding of caregiver burden, as well as helping guide the development and implementation of appropriate support services for this important and under-represented caregiver population. The current study therefore aims to add to the general literature this breadth of information, as well as help address a gap in the literature concerning the effects of AD caregiving on Japanese caregivers’ work productivity and resource use, an issue of great relevance in the context of a rapidly aging population [48]. The study will assess how family caregivers for patients with AD or dementia in Japan differed from non-caregivers in terms of their characteristics and health outcomes (i.e., comorbidities, health-related quality of life [HRQoL], productivity, and resource use).

## Methods

### Sample

This study analyzed existing data from caregivers identified in the 2012 and 2013 Japan National Health and Wellness Surveys (NHWS; total *n* = 60000). NHWS is a self-administered, self-reported, Internet-based questionnaire of a sample of adults (aged 18 or older), collecting sociodemographic and general health history information, as well as information on comorbid conditions, medication usage, and health outcomes, etc. Stratified samples (by sex and age) were implemented to ensure demographic compositions representative of corresponding adult populations. Respondents were originally selected from opt-in consumer panels; see further details about the panel recruitment in a very similar study and analysis using Brazil data [33], as well as details about the Japan panel in particular and how well it aligned with census data regarding gender, age, income, and region [49]. NHWS respondents needed to read and write in Japanese and to provide their informed consent in order to participate in the Japan NHWS. The NHWS protocol and questionnaire were reviewed and approved by Essex Institutional Review Board (Lebanon, New Jersey, USA). Other published studies have also used NHWS Japan data, with additional details provided on incentives and translation procedures [49–51].

Caregivers were defined as those who reported currently caring for an adult relative, with “Alzheimer’s disease or dementia” selected among fifteen prompted conditions (*n* = 1302). Non-caregivers were defined as those not currently caring for an adult relative with any of the other prompted conditions or any unprompted condition (*n* = 53758). Excluded from analyses were caregivers for adult relatives with any other condition, as well as 2012 data from respondents who participated in both years of NHWS (i.e., only 2013 data were retained to avoid double-counting respondents).

### Measures

#### Baseline comorbidities

The Charlson comorbidity index (CCI; [52]) weights the presence of several mortality-predicting conditions and sums the result, with higher total index scores indicating greater comorbid burden on the patient. In the re-contact study, the Quan et al. [53] scoring of CCI was used, providing an updated scoring algorithm based on fewer conditions (congestive heart failure, dementia, chronic pulmonary disease, rheumatologic disease, mild liver disease, diabetes with end organ damage, hemiplegia, moderate/severe renal disease, metastatic tumor, acquired immune deficiency syndrome/human immunodeficiency virus, and lymphoma, leukemia, or any tumor) and reflecting weighting from a more recent replication. CCI scores were assessed as covariates, not as outcomes of interest, given their likely role as pre-occurring conditions to caregiving. The greater the total index score (ranging from 0 to 24 theoretically), the greater the comorbid burden on the caregiver. Paraplegia and moderate/severe liver disease were not assessed in NHWS and were therefore excluded from the scoring (with a corresponding maximum score of 22).

#### Sociodemographics, health characteristics and behaviors

In NHWS, additional variables were compared across caregivers vs. non-caregivers, including: sex, age, marital status, employment, income, education, children in household, smoking, drinking alcohol, exercise, body mass index (BMI), and health insurance.

### Comorbidities

Select comorbidities were examined to assess the comorbid burden associated with caregiving. Self-reported diagnosis “by a physician” with depression, anxiety, insomnia, hypertension, pain, and diabetes (Type 1/2) were assessed as outcomes of interest, given their potential role as consequences of caregiving, or as conditions exacerbated by caregiving.

### Depression

The Patient Health Questionnaire (PHQ-9; [54, 55]) measures frequency of depression symptoms, with items scored on a 4-point scale (not at all = 0 to nearly every day = 3). A cut-off of ≥ 10 vs. < 10 [56], suggestive of major depressive disorder (MDD), was used in addition to the “diagnosed depression” measure.

### Work productivity

Productivity impairments were assessed using the four metrics of the Work Productivity and Activity Impairment (WPAI) questionnaire [57, 58], with respect to one’s health in the past seven days. Absenteeism was quantified as percentage of work time missed, based on reported hours missed divided by hours worked plus missed. Presenteeism was based on reported percentage impairment experienced while at work. Overall work productivity loss was quantified as a percentage combining absenteeism plus presenteeism multiplied by percentage time worked. Activity impairment was based on reported percentage impairment in daily activities. Only respondents who reported being full-time, part-time, or self-employed provided data for absenteeism, presenteeism, and overall work impairment. Among all respondents, WPAI assessed the effect of their health on daily activities. Higher percentage values indicate greater impairment.

### HRQoL

Short Form (SF)-36v2-derived scores [59], including Physical (PCS) and Mental (MCS) Component Summary scores, normed to a mean of 50 and standard deviation of 10 based on the US population, along with health state utilities derived via the SF-6D algorithm [60], were used to assess the overall burden associated with caregiving. Higher scores indicate better health status, and differences exceeding 3 points (PCS and MCS) or 0.041 points (SF-6D health utilities) are considered minimally important differences (MIDs; [59, 61]).

### Healthcare resource utilization

Self-reported resource use, independent of a specific cause, was defined by the number of traditional healthcare provider visits, emergency room (ER) visits, and hospitalizations for one’s medical condition in the past 6 months, as well as frequency of any (1+ vs. 0) visits.

### Analysis

#### Descriptive analyses

Descriptive results were examined for all study variables of interest, including sample sizes and proportions for categorical variables and means, standard deviations (SDs), ranges, and percentiles for continuous variables. Demographics and health characteristics were analyzed for the total sample, to identify the characteristics of caregivers in Japan, as well as to identify the characteristics of non-caregivers.

### Bivariate analyses

Significant differences were assessed among caregivers for patients with AD or dementia, compared with non-caregivers, using two - sample binomial proportion and Chi-square tests of independence (frequencies) or independent sample t tests (means). Caregiver demographics and health characteristics were compared first, in order to understand baseline differences and inform variables included as covariates in multivariable analysis.

### Multivariable analyses

Given initial evidence of baseline discrepancies between caregivers and non-caregivers, propensity matching was used to control for confounding. A binary logistic regression model, predicting caregiver vs. non-caregiver status as a function of all potential baseline variables (sex, age, BMI, exercise, alcohol, smoking, marital status, CCI, insured status, education, employment, income, and children in household), was used to generate propensity scores. These scores represent the propensity of inclusion in one group vs. the other, based on an individual’s values on all the covariates. Caregivers were each then matched with non-caregivers who had similar probabilities of having been caregivers, according to their scores. Bivariate comparisons on this select subset of caregivers and non-caregivers were used to provide an estimate of how health outcomes differ between these populations. The one-to-one matching was conducted using a “greedy matching” algorithm, excluding respondents who did not have an exact match within the specified level of precision.

## Results

Among 60000 total 2012 + 2013 NHWS respondents, 1302 unique (i.e., non-duplicated) caregivers for an adult relative with AD or dementia were compared against 53758 non-caregivers (i.e., those not currently caring for an adult relative with any condition), total *n* = 55060. Caregivers in Japan were on average 52.6 years old, and 53.0 % were female, 68.2 % married or living with a partner, 98.5 % insured, and 20.7 % living with one or more children in the household (see Table 1).

**Table 1:** Sociodemographics and health characteristics as a function of caregiver status

		Non-caregivers (*n* = 53758)		Caregivers (*n* = 1302)		Total (*n* = 55060)	
		Mean/%	SD/n	Mean/%	SD/n	Mean/%	SD/n
Age of the respondent: mean		47.53	15.66	52.63^*^	13.89	47.65	15.64
Age: category	18–29	17.1 %	9191	11.2 %	146^*^	17.0 %	9337
	30–39	18.7 %	10045	8.8 %	114^*^	18.5 %	10159
	40–49	17.9 %	9635	10.8 %	140^*^	17.8 %	9775
	50–64	25.6 %	13765	46.2 %	602^*^	26.1 %	14367
	≥65	20.7 %	11122	23.0 %	300^*^	20.7 %	11422
Gender	Female	49.2 %	26425	53.0 %	690^*^	49.2 %	27115
	Male	50.8 %	27333	47.0 %	612^*^	50.8 %	27945
BMI category	Underweight	11.0 %	5910	9.8 %	128	11.0 %	6038
	Normal weight	67.9 %	36486	68.7 %	894	67.9 %	37380
	Overweight	14.7 %	7909	16.4 %	214	14.8 %	8123
	Obese	2.7 %	1433	3.1 %	41	2.7 %	1474
	Unknown	3.8 %	2020	1.9 %	25^*^	3.7 %	2045
Exercise frequency (20+ minutes) in past month	0–11 times	83.6 %	44933	80.6 %	1050^*^	83.5 %	45983
	≥12 times	16.4 %	8825	19.4 %	252^*^	16.5 %	9077
Alcohol use per week	≤ once	61.6 %	33131	54.6 %	711^*^	61.5 %	33842
	≥2–3 times	38.4 %	20627	45.4 %	591^*^	38.5 %	21218
Smoke cigarettes	No	79.7 %	42842	74.9 %	975^*^	79.6 %	43817
	Yes	20.3 %	10916	25.1 %	327^*^	20.4 %	11243
Marital status	Single	30.2 %	16252	22.9 %	298^*^	30.1 %	16550
	Married/living with partner	62.4 %	33519	68.2 %	888^*^	62.5 %	34407
	Divorced/separated/widowed > 1 year	7.0 %	3777	8.2 %	107	7.1 %	3884
	Divorced/separated/widowed ≤ 1 year	0.4 %	210	0.7 %	9	0.4 %	219
CCI: mean score		0.136	0.47	0.369^*^	1.67	0.14	0.53
CCI: category	0	89.3 %	48022	80.8 %	1052^*^	89.1 %	49074
	1	8.5 %	4556	13.5 %	176^*^	8.6 %	4732
	2	1.7 %	906	3.1 %	40^*^	1.7 %	946
	>3	0.5 %	274	2.6 %	34^*^	0.6 %	308
Health insurance	None of the above	3.2 %	1731	1.5 %	19^*^	3.2 %	1750
	National/Social/Late Stage Elderly	96.8 %	52027	98.5 %	1283^*^	96.8 %	53310
Types of current medical insurance	National Health Insurance	44.9 %	24142	50.1 %	652^*^	45.0 %	24794
	Social Insurance	48.7 %	26207	45.9 %	598^*^	48.7 %	26805
	Late Stage Elderly Insurance	1.5 %	780	1.1 %	14	1.4 %	794
	Other	1.7 %	898	1.5 %	19	1.7 %	917
	None of the above	3.2 %	1731	1.5 %	19^*^	3.2 %	1750
Education: college or above	High school or lower	36.6 %	19651	33.3 %	433^*^	36.5 %	20084
	College or above	63.4 %	34107	66.7 %	869^*^	63.5 %	34976
Highest level of education completed or highest degree received	Elementary School	0.2 %	121	0.3 %	4	0.2 %	125
	Junior High School	2.5 %	1346	2.0 %	26	2.5 %	1372
	High School	33.8 %	18184	31.0 %	403^*^	33.8 %	18587
	2 Year College	15.6 %	8385	15.9 %	207	15.6 %	8592
	4 Year College	42.7 %	22966	45.2 %	588	42.8 %	23554
	Graduate School	5.1 %	2756	5.7 %	74	5.1 %	2830
Employment status	Not employed, disabled, retired, student, or homemaker	40.5 %	21790	43.8 %	570^*^	40.6 %	22360
	Full-time, part-time, or self-employed	59.5 %	31968	56.2 %	732^*^	59.4 %	32700
Income	< ¥3,000,000	18.6 %	10012	18.0 %	234	18.6 %	10246
	¥3,000,000 to < ¥5,000,000	26.1 %	14014	23.3 %	303^*^	26.0 %	14317
	¥5,000,000 to < ¥8,000,000	25.2 %	13564	25.5 %	332	25.2 %	13896
	≥ ¥8,000,000	20.3 %	10937	27.3 %	356^*^	20.5 %	11293
	Declined to answer	9.7 %	5231	5.9 %	77^*^	9.6 %	5308
Children (<18) in household	0	75.1 %	40376	79.3 %	1032^*^	75.2 %	41408
	1	13.1 %	7038	12.3 %	160	13.1 %	7198
	2	9.5 %	5125	6.6 %	86^*^	9.5 %	5211
	≥3	2.3 %	1219	1.8 %	24	2.3 %	1243

Caregivers were defined as those currently caring for an adult relative with Alzheimer’s disease or dementia, whereas non-caregivers were those not currently caring for an adult relative with any listed condition

*BMI* body mass index, *CCI* Charlson comorbidity index, *SD* standard deviation

*p < 0.05 for the two-tailed significance of differences across caregivers and non-caregivers within a given row

With respect to baseline characteristics, caregivers, compared with non-caregivers, were on average older, more frequently female, married/partnered, frequent alcohol drinkers, current smokers, and exercisers, and they had higher average CCI scores and were less likely to be employed. Caregivers vs. non-caregivers were also more likely to have health insurance, greater education, higher income, and fewer children in the household.

### Full sample comparisons

#### Comorbidities

Caregivers vs. non-caregivers experienced greater depression, whether demonstrated in higher PHQ-9 scores indicating greater severity, greater frequency of MDD according to the PHQ-9, or greater likelihood of self-reported diagnosis with depression (Table 2). Caregivers vs. non-caregivers also reported more frequent insomnia, anxiety, hypertension, pain, and diabetes.

**Table 2:** Health outcomes as a function of caregiver status

		Non-caregivers (*n* = 53758)		Caregivers (*n* = 1302)		Total (*n* = 55060)	
		Mean/%	SD/n	Mean/%	SD/n	Mean/%	SD/n
PHQ-9		3.18	4.75	4.40^*^	5.48	3.21	4.77
PHQ-9: category	PHQ-9: < 10	91.1 %	48957	85.8 %	1117^*^	90.9 %	50074
	PHQ-9: ≥ 10 (MDD)	8.9 %	4801	14.2 %	185^*^	9.1 %	4986
Diagnosed comorbidities							
	Depression	3.3 %	1778	6.2 %	81^*^	3.4 %	1859
	Insomnia	4.4 %	2361	9.8 %	128*	4.5 %	2489
	Anxiety	0.8 %	448	2.0 %	26*	0.9 %	474
	Hypertension	11.7 %	6290	17.5 %	228*	11.8 %	6518
	Pain	7.9 %	4269	15.5 %	202*	8.1 %	4471
	Diabetes (Type 1/2)	3.7 %	1981	6.1 %	79*	3.7 %	2060
SF-6D		0.765	0.121	0.724*	0.124	0.764	0.121
SF-36v2: Physical Component Summary		53.57	6.09	51.58*	6.62	53.52	6.11
SF-36v2: Mental Component Summary		48.01	9.61	46.00*	10.65	47.96	9.64
WPAI: % absenteeism: work missed due to health^a^		2.87	12.38	5.38*	15.79	2.93	12.47
WPAI: % presenteeism-related impairment: impairment while working due to health^a^		18.64	23.21	22.75*	25.42	18.73	23.27
WPAI: % overall work impairment due to health^a^		20.26	25.23	25.69*	28.16	20.38	25.31
WPAI: % activity impairment due to health		20.71	24.44	25.43*	25.82	20.83	24.49
Emergency room visits, past 6 months		0.08	0.89	0.29*	1.78	0.09	0.92
Hospitalizations, past 6 months		0.46	4.09	0.83*	5.23	0.47	4.12
Traditional healthcare provider visits, past 6 months		4.38	7.70	7.74*	18.53	4.46	8.14
Emergency room visits (1+), past 6 months		3.2 %	1722	7.3 %	95*	3.3 %	1817
Hospitalizations (1+), past 6 months		4.0 %	2152	8.2 %	107*	4.1 %	2259
Traditional healthcare provider visits (1+), past 6 months		56.5 %	30388	74.9 %	975*	57.0 %	31363

Caregivers were defined as those currently caring for an adult relative with Alzheimer’s disease or dementia, whereas non-caregivers were those not currently caring for an adult relative with any listed condition

*MDD* major depressive disorder, *PHQ* patient health questionnaire, *SD* standard deviation, *SF* short form, *WPAI* work productivity and activity impairment

*p < 0.05 for the two-tailed significance of differences across caregivers and non-caregivers within a given row

^a^Absenteeism and overall work impairment results were available only for employed respondents, excluding those who both worked and missed 0 h, as well as outliers who reported > 112 potential work hours in the past week (*n* = 30737: 693 caregivers and 30044 non-caregivers). Presenteeism-related impairment results were available only for employed respondents who worked >0 h in the past week (*n* = 31460: 712 caregivers and 30748 non-caregivers)

### HRQoL

Caregivers vs. non-caregivers experienced significantly lower health utilities, reaching the MID for meaningfully poorer HRQoL. Caregivers also had significantly lower PCS and MCS scores, indicating poorer physical and mental health status, respectively (Table 2).

### Work productivity

Caregivers vs. non-caregivers reported significantly higher activity impairment, as well as (among employed respondents only) greater absenteeism, presenteeism-related impairment, and overall work impairment (Table 2).

### Resource use

Caregivers vs. non-caregivers reported significantly more visits to the ER, hospital, and healthcare providers in the past 6 months (Table 2).

### Matched comparisons

#### Comorbidities

With one-to-one propensity matching, where 1297 out of 1302 caregivers were successfully matched against 1297 non-caregivers, many comorbidities remained significantly different across the two groups (Table 3). Namely, caregivers vs. non-caregivers experienced greater depression, based on higher PHQ-9 scores (4.4 vs. 3.1), more frequent PHQ-9 based MDD (14.2 % vs. 8.6 %), and more frequent self-reported diagnosed depression (6.2 % vs. 3.2 %), all *p* < 0.05. Caregivers vs. non-caregivers also reported more frequent insomnia (9.8 % vs. 5.7 %), anxiety (1.9 % vs. 0.4 %), and pain (15.5 % vs. 10.6 %), all *p* < 0.05. Hypertension and diabetes no longer differed significantly.

**Table 3:** Health outcomes as a function of caregiver status across propensity-matched groups

		Non-caregivers (*n* = 1297)		Caregivers (*n* = 1297)		Total (*n* = 2594)	
		Mean/%	SD/n	Mean/%	SD/n	Mean/%	SD/n
PHQ-9		3.09	4.68	4.39*	5.47	3.74	5.13
PHQ-9: category	PHQ-9: < 10	91.4 %	1185	85.8 %	1113*	90.9 %	2298
	PHQ-9: ≥ 10 (MDD)	8.6 %	112	14.2 %	184*	9.1 %	296
Diagnosed comorbidities							
	Depression	3.2 %	42	6.2 %	80*	3.4 %	122
	Insomnia	5.7 %	74	9.8 %	127*	4.5 %	201
	Anxiety	0.4 %	5	1.9 %	25*	0.9 %	30
	Hypertension	17.3 %	225	17.4 %	226	11.8 %	451
	Pain	10.6 %	138	15.5 %	201*	8.1 %	339
	Diabetes (Type 1/2)	7.9 %	103	6.1 %	79	3.7 %	182
SF-6D		0.764	0.123	0.724*	0.124	0.744	0.125
SF-36v2: Physical Component Summary		52.73	6.71	51.60*	6.61	52.17	6.69
SF-36v2: Mental Component Summary		48.60	9.52	46.00*	10.65	47.30	10.18
WPAI: % absenteeism: work missed due to health^a^		3.08	12.30	5.40*	15.81	4.25	14.23
WPAI: % presenteeism-related impairment: impairment while working due to health^a^		18.44	23.83	22.74*	25.39	20.62	24.72
WPAI: % overall work impairment^a^		20.36	25.66	25.75*	28.18	23.09	27.09
WPAI: % activity impairment		21.79	25.37	25.41*	25.82	23.60	25.66
Emergency room visits, past 6 months		0.08	0.56	0.27*	1.68	0.18	1.26
Hospitalizations, past 6 months		0.81	6.72	0.79	5.15	0.80	5.99
Traditional healthcare provider visits, past 6 months		5.35	8.59	7.70*	18.50	6.53	14.47
Emergency room visits (1+), past 6 months		4.0 %	52	7.2 %	94*	3.3 %	146
Hospitalizations (1+), past 6 months		5.1 %	66	8.1 %	105*	4.1 %	171
Traditional healthcare provider visits (1+), past 6 months		61.9 %	803	74.9 %	971*	57.0 %	1774

Caregivers were defined as those currently caring for an adult relative with Alzheimer’s disease or dementia, whereas non-caregivers were those not currently caring for an adult relative with any listed condition

*MDD* major depressive disorder, *PHQ* patient health questionnaire, *SD* standard deviation, *SF* short form, *WPAI* work productivity and activity impairment

*p < 0.05 for the two-tailed significance of differences across caregivers and non-caregivers within a given row

^a^Absenteeism and overall work impairment results were available only for employed respondents, excluding those who both worked and missed 0 h, as well as outliers who reported > 112 potential work hours in the past week (*n* = 1364: 691 caregivers and 673 non-caregivers). Presenteeism-related impairment results were available only for employed respondents who worked > 0 h in the past week (*n* = 1397: 709 caregivers and 688 non-caregivers)

### HRQoL

Matched caregivers vs. non-caregivers experienced significantly lower health utilities (0.724 vs. 0.764), *p* < 0.05, nearly reaching the MID for meaningfully poorer HRQoL (Table 3). Caregivers also had significantly lower PCS (51.60 vs. 52.73) and MCS (46.00 vs. 48.60), both *p* < 0.05.

### Work productivity

Matched caregivers vs. non-caregivers reported significantly higher activity impairment (25.4 % vs. 21.8 %), as well as greater absenteeism (5.4 % vs. 3.1 %), presenteeism-related impairment (22.7 % vs. 18.4 %), and overall work impairment (25.8 % vs. 20.4 %), all *p* < 0.05 (Table 3).

### Resource use

Matched caregivers vs. non-caregivers reported significantly more visits to the ER (0.27 vs. 0.08) and healthcare providers (7.70 vs. 5.35) in the past 6 months, both *p* < 0.05, but not significantly more hospital visits (0.79 vs. 0.81). Matched caregivers vs. non-caregivers also reported more frequently any visits to the ER (7.2 % vs. 4.0 %), hospital (8.1 % vs. 5.1 %), and healthcare providers (74.9 % vs. 61.9 %), all *p* < 0.05 (Table 3).

## Discussion

The diagnosis of a terminal or chronic illness can often result in family members being required to provide informal care to patients. Whereas this form of caregiving can have positive effects through benefit-finding and meaning assigned to caring [21–24] across a variety of diseases, it can also be associated with significant burden in physical and psychosocial domains. In the current study, significant burden was identified among individuals providing informal care for those diagnosed with AD or dementia. These findings are aligned with previous studies both in Japan and globally, suggesting that the burden experienced by caregivers extends across geographies, in spite of any influence of individual healthcare systems or cultural differences. Moreover, given little research in Japan investigating this form of burden or exploring its effects on health and economic outcomes, the current study identified potential impact of caregiving not only on quality of life, but also on comorbidities, productivity, and use of healthcare resources.

The caregivers recruited in the current study were on average 52.6 years old, somewhat younger than those involved in other studies of AD (e.g. 63.8 years [45]); however, as in the current study, the majority of caregivers in previous studies were female [45]. The age difference may be representative of the Internet-based format of this study, which may recruit younger individuals more comfortable with the online format.

The current study found that across a majority of health outcome measures, caregivers experienced significantly greater burden than did non-caregivers, even after matching them against non-caregivers with very similar baseline characteristics. Caregivers experienced greater frequency of comorbidities, including depression, insomnia, anxiety, and pain compared with non-caregivers.

Compared with non-caregivers, caregivers reported significantly lower health utilities, nearly reaching the MID for meaningfully poorer HRQoL, and they had significantly poorer physical and mental health status. Similar associations have been reported in the global literature, including among caregivers in the UK and Europe [62–64], thus reinforcing the critical importance of providing support to this vulnerable group.

Further, significant economic consequences were found among study caregivers compared with matched controls. These individuals reported significantly higher work productivity and activity impairment across all metrics. Finally, caregivers reported significantly greater resource use: i.e., more visits to the ER and healthcare providers in the previous six months and more frequent reports of any visit to the ER, hospital, or healthcare providers, further reinforcing the multifaceted economic impact of caregiver burden, as noted in previous studies outside of Japan [25]. With respect to baseline characteristics, caregivers had relatively poorer health and greater comorbid risk than (all, i.e., unmatched) non-caregivers, but they also had more financial resources at their disposal, lack of employment notwithstanding.

The current results align with previous global caregiver studies, including in particular a recent, similar analysis of burden among 209 caregivers of AD patients in NHWS Brazil [33]. For example, relative to their corresponding non-caregiver peers, the Japanese caregivers in the current study experienced health utilities impairments (−0.040 points) comparable to those of the Brazil caregivers (−0.024), as well as comparable impairments on PCS (−1.13 in Japan vs. -0.94 in Brazil) and MCS (−2.60 vs. -1.70, respectively), overall work impairment (26.5 % greater impairment in Japan vs. 35.9 % greater in Brazil), absenteeism (75.3 % vs. 59.6 % greater, respectively), presenteeism-related impairment (23.3 % vs. 32.7 % greater), activity impairment (16.6 % vs. 16.7 % greater), provider visits (43.9 % vs. 28.7 % greater), depression (odds ratio [OR] of 2.0 in Japan vs. 2.0 in Brazil), MDD (OR = 1.8 vs. 1.5, respectively), anxiety (OR = 4.8 vs. 1.7), insomnia (OR = 1.8 vs. 1.6), and pain (OR = 1.5 vs. 1.7).

The impairments found in the current study were also comparable to those reported by caregivers for adult relatives diagnosed with cancer in yet another NHWS-based study in the EU, where health utilities were 0.043 points lower for caregivers vs. non-caregivers (vs. 0.040 points lower for Japanese caregivers in the current study), PCS and MCS were 1.29 and 3.26 points lower, respectively (vs. 1.13 and 2.60 in the current study), overall work impairment, absenteeism, presenteeism-related impairment, and activity impairment were 46, 69, 45, and 32 % greater, respectively (vs. 26 %, 75 %, 23 %, and 17 % greater), provider visits were 33.2 % greater (vs. 43.9 %), and depression, anxiety, and insomnia had ORs = 1.5, 2.0, and 2.0, respectively (vs. 2.0, 4.8, and 1.8) [65].

In yet another NHWS-based study in the US, the burden of caregiving for patients with AD was compared with that for patients with multiple sclerosis (MS), finding that impairments did not differ significantly across the two conditions in terms of work productivity measures, HRQoL, or provider visits, although activity impairment, ER visits, and hospitalizations were significantly greater for caregivers of patients with MS [66].

It is important to note that the comparisons of caregiver burden referenced above are with respect to general health outcomes domains: mental health-related comorbidities, overall mental and physical status, productivity impairments, and resource use. There may be other, more disease-specific variations in burden that were not captured in the current or comparable studies. For example, the specific needs and psychological experiences of caring for individuals with severe dementia or AD may differ from those of caregiving in other conditions, even as general health outcome impairments are similar in degree. Generally, with variations in terms of magnitude and significance of differences across health outcomes, the current study finds that caregivers for patients with AD in Japan experienced impairments comparable to those experienced by caregivers for patients with AD in other geographies (e.g., Brazil and US), as well as impairments experienced by caregivers for patients with other conditions (cancer, MS, and schizophrenia) across geographies [65–67].

### Strengths and limitations

The online survey format provides certain sampling advantages, including a broad representation of the overall population (including alignment with census data for Japan) and wide variance in caregiver and non-caregiver characteristics, but it may under-represent caregivers without access to or comfort with online technology (i.e., those who are not “digitally literate”). This online format may also bias recruitment toward younger, wealthier segments of society. Despite this, the fact that significant impairment and burden was established even among this group of caregivers suggests that under-represented groups (older, resource poor) may experience even greater burden than what was found in the current study.

While this study provides important insight into the potential health and economic effects of caregiving in the context of AD, the cross-sectional study design precludes any definitive conclusions from being drawn about causal relationships. Moreover, the self-report nature of the NHWS provides the potential for recall and other reporting biases (e.g., imprecise or inaccurate estimates of time, past productivity, and past resource use).

The NHWS did not collect information regarding the relationship status between patients and their caregivers, nor did it collect information on the level or type of care being provided. Many caregivers may have been the offspring of patients, as suggested by the relatively young age of caregivers in the current study. To the extent that this was the case, AD may have a meaningful impact on the work productivity of younger caregivers in the working population. It should be noted that the caregiving relationship can be complex and shift over time, influenced by both patient and caregiver characteristics, external pressures, and the availability of financial and other resources. Future research should thus clarify caregiver-patient relationships and surrounding issues, further elucidating the role that family members assume in caring for those with AD, which can inform the provision of more effective supportive services.

Moreover, the NHWS did not provide data on the precise proportion of caregivers who cared for patients with DAT vs. other forms of dementia. While research suggests that AD composes a majority of dementia among patients, it is unclear how this translates into caregiving (e.g., whether caregiving is more prevalent among AD patients). Also, the NHWS did not provide data on patients’ disease progression and severity, and patients with more severe disease are expected to have correspondingly older caregivers and poorer patient outcomes [25, 45]. Therefore, future research should assess the specific contributions of different types of dementia or disease severity to types and degree of caregiver burden.

Finally, the NHWS did not provide detailed data on non-caregivers’ family relationships, meaning that the incremental burden observed in caregivers vs. non-caregivers may in some part have been caused by concerns relating merely to having an older relative with or without dementia, regardless of caregiving status. At the same time, the propensity matching employed in the study (especially controlling for variables such as age, employment and marital status, and children in the household) should have limited considerably the heterogeneity among non-caregivers and provided a relatively focused comparison against a pool of matched non-caregivers similar to the caregivers.

Additional studies are needed to better understand those who are non-caregivers, particularly for the subset of persons who have a relative with dementia but have not taken on caregiver responsibilities. This research may help elucidate how and why people become caregivers and the impact of not being able to fulfill the role of caregiver.

### Implications

The current study reinforces understanding of the significant health-related burden that AD caregivers can experience, extending this research and quantifying the impact family caregiving can have not only on quality of life but also on comorbidities, work productivity, and healthcare resource use. Supportive care programs for caregivers often rely upon traditional counseling modalities, requiring time away from caregiving responsibilities and potentially hampered by stigma surrounding mental health. The potential for family caregiving to impair work productivity could therefore suggest innovative worksite-based interventions and provide a preliminary basis for gaining employer support. Such interventions could provide both instrumental (e.g., time to run errands) and emotional support in a less stigma-prone environment, while reducing time spent away from caregiving responsibilities and mitigating the negative impact of caregiving on work productivity. At the same time, interventions focused on screening and treatment of depressive symptoms may offer another modality via which to identify and reach out to caregivers, perhaps focusing on strengthening the positive aspects of caregiving to better address the unique needs of this population.

## Conclusions

Those providing care for patients with dementia (including dementia due to AD) in Japan experience a broad range of care-related burden (physical, psychological, social, and financial), with relatively greater comorbid risk, poorer HRQoL, greater productivity impairment, and higher rates of healthcare resource use. Given aging world populations and corresponding projected increases in AD/dementia diagnoses over time, direct treatment and policies to help caregivers and their patients are needed. Special consideration may need to be given to interventions related to work environments and screening/treatment of depression, given productivity impairments and comorbidities identified among the range of health outcomes assessed in the current study. With Japan’s demographic changes leading to a relatively small workforce supporting the care and financial needs of a growing elderly population, policymakers need to consider the impact of caregiving on many aspects of society. The current study helps to better characterize caregivers in order to help identify where support is most needed. Further research in Japan can help elucidate the broader impact of AD on caregivers and society in general, including assessments of how the caregiver experience is influenced by AD severity and current forms of assistance (e.g., private care and insurance programs), as well as assessments of interventions to assist caregivers.

## Abbreviations

AD, Alzheimer’s disease; BMI, body mass index; CCI, Charlson comorbidity index; DAT, dementia of the Alzheimer’s type; ER, emergency room; HRQoL, health-related quality of life; MCS, mental component summary; MDD, major depressive disorder; MID, minimally important difference; MMSE, mini-mental state examination; MS, multiple sclerosis; NHWS, National Health and Wellness Survey; OR, odds ratio; PCS, physical component summary; PHQ, patient health questionnaire; SD, standard deviation; SF, short form; WPAI, work productivity and activity impairment.

## References

[1] National Institute on Aging. Alzheimer’s Disease Fact Sheet. 2015. http://www.nia.nih.gov/alzheimers/publication/alzheimers-disease-fact-sheet. Accessed October 15 2015.

[2] Association A’s (2014). 2014 Alzheimer’s Disease Facts and Figures. Alzheimers Dement.

[3] Brookmeyer R, Johnson E, Ziegler-Graham K, Arrighi HM (2007). Forecasting the global burden of Alzheimer’s disease. Alzheimers Dement.

[4] Carter R (2008). Addressing the caregiving crisis. Prev Chronic Dis.

[5] Hebert LE, Weuve J, Scherr PA, Evans DA (2013). Alzheimer disease in the United States (2010–2050) estimated using the 2010 census. Neurology.

[6] Duthey B. Background paper 6.11: Alzheimer disease and other dementias. A Public Health Approach to Innovation. 2013. [(accessed on 7 June 2016)]. pp. 1–74. Update on 2004 Background Paper. Availableonline: http://www.who.int/medicines/areas/priority_medicines/BP6_11Alzheimer.pdf.

[7] Asada T (2012). Prevalence of dementia in Japan: past, present and future. Rinsho Shinkeigaku.

[8] Kasai M, Nakamura K, Meguro K (2010). Alzheimer’s disease in Japan and other countries: review of epidemiological studies in the last 10 years. Brain Nerve.

[9] Meguro K, Ishii H, Yamaguchi S, Ishizaki J, Shimada M, Sato M (2002). Prevalence of dementia and dementing diseases in Japan: the Tajiri project. Arch Neurol.

[10] Sekita A, Ninomiya T, Tanizaki Y, Doi Y, Hata J, Yonemoto K (2010). Trends in prevalence of Alzheimer’s disease and vascular dementia in a Japanese community: the Hisayama Study. Acta Psychiatr Scand.

[11] Dodge HH, Buracchio TJ, Fisher GG, Kiyohara Y, Meguro K, Tanizaki Y (2012). Trends in the prevalence of dementia in Japan. Int J Alzheimers Dis.

[12] Chaudhuri JD, Das S (2006). The Role of Caregivers in the Management of Alzheimer’s disease: Examples from Asian Countries. Sultan Qaboos Univ Med J.

[13] Yamada T, Hattori H, Miura A, Tanabe M, Yamori Y (2001). Prevalence of Alzheimer’s disease, vascular dementia and dementia with Lewy bodies in a Japanese population. Psychiatry Clin Neurosci.

[14] Sugino H, Watanabe A, Amada N, Yamamoto M, Ohgi Y, Kostic D (2015). Global Trends in Alzheimer Disease Clinical Development: Increasing the Probability of Success. Clin Ther.

[15] Nakanishi M, Nakashima T (2014). Features of the Japanese national dementia strategy in comparison with international dementia policies: How should a national dementia policy interact with the public health- and social-care systems?. Alzheimers Dement.

[16] Moore MJ, Zhu CW, Clipp EC (2001). Informal costs of dementia care: estimates from the National Longitudinal Caregiver Study. J Gerontol B Psychol Sci Soc Sci.

[17] Brodaty H, Donkin M (2009). Family caregivers of people with dementia. Dialogues Clin Neurosci.

[18] Hearson B, McClement S (2007). Sleep disturbance in family caregivers of patients with advanced cancer. Int J Palliat Nurs.

[19] Schulz R, Beach SR (1999). Caregiving as a risk factor for mortality: the Caregiver Health Effects Study. JAMA.

[20] Schulz R, O’Brien AT, Bookwala J, Fleissner K (1995). Psychiatric and physical morbidity effects of dementia caregiving: prevalence, correlates, and causes. Gerontologist.

[21] Cassidy T (2013). Benefit finding through caring: the cancer caregiver experience. Psychol Health.

[22] Cheng ST, Lau RW, Mak EP, Ng NS, Lam LC (2014). Benefit-finding intervention for Alzheimer caregivers: conceptual framework, implementation issues, and preliminary efficacy. Gerontologist.

[23] Kim Y, Schulz R, Carver CS (2007). Benefit-finding in the cancer caregiving experience. Psychosom Med.

[24] Lum HD, Lo D, Hooker S, Bekelman DB (2014). Caregiving in heart failure: relationship quality is associated with caregiver benefit finding and caregiver burden. Heart Lung.

[25] Wimo A, Reed CC, Dodel R, Belger M, Jones RW, Happich M (2013). The GERAS Study: a prospective observational study of costs and resource use in community dwellers with Alzheimer’s disease in three European countries--study design and baseline findings. J Alzheimers Dis.

[26] Sado M. Research on the economical impact for dementia in Japan, 2013–2014 comprehensive study report: MHLW grant-in-aid for scientific research 2014.

[27] Bergvall N, Brinck P, Eek D, Gustavsson A, Wimo A, Winblad B (2011). Relative importance of patient disease indicators on informal care and caregiver burden in Alzheimer’s disease. Int Psychogeriatr.

[28] Fuller-Jonap F, Haley WE (1995). Mental and physical health of male caregivers of a spouse with Alzheimer’s disease. J Aging Health.

[29] Kolanowski AM, Fick D, Waller JL, Shea D (2004). Spouses of persons with dementia: their healthcare problems, utilization, and costs. Res Nurs Health.

[30] Lu YF, Wykle M (2007). Relationships between caregiver stress and self-care behaviors in response to symptoms. Clin Nurs Res.

[31] Beeson R, Horton-Deutsch S, Farran C, Neundorfer M (2000). Loneliness and depression in caregivers of persons with Alzheimer’s disease or related disorders. Issues Ment Health Nurs.

[32] Langa KM, Chernew ME, Kabeto MU, Herzog AR, Ofstedal MB, Willis RJ (2001). National estimates of the quantity and cost of informal caregiving for the elderly with dementia. J Gen Intern Med.

[33] Laks J, Goren A, Duenas H, Novick D, Kahle-Wrobleski K (2015). Caregiving for patients with Alzheimer’s disease or dementia and its association with psychiatric and clinical comorbidities and other health outcomes in Brazil. Int J Geriatr Psychiatry.

[34] Arai A, Matsumoto T, Ikeda M, Arai Y (2007). Do family caregivers perceive more difficulty when they look after patients with early onset dementia compared to those with late onset dementia?. Int J Geriatr Psychiatry.

[35] Arai Y (2005). Medical and nursing care of dementia patients. Nursing burden of family caregivers. Nihon Naika Gakkai Zasshi.

[36] Arai Y, Kumamoto K, Washio M, Ueda T, Miura H, Kudo K (2004). Factors related to feelings of burden among caregivers looking after impaired elderly in Japan under the Long-Term Care insurance system. Psychiatry Clin Neurosci.

[37] Arai Y, Zarit SH (2011). Exploring strategies to alleviate caregiver burden: effects of the National Long-Term Care insurance scheme in Japan. Psychogeriatrics.

[38] Kurasawa S, Yoshimasu K, Washio M, Fukumoto J, Takemura S, Yokoi K (2012). Factors influencing caregivers’ burden among family caregivers and institutionalization of in-home elderly people cared for by family caregivers. Environ Health Prev Med.

[39] Sasaki M, Arai Y, Kumamoto K, Abe K, Arai A, Mizuno Y (2007). Factors related to potentially harmful behaviors towards disabled older people by family caregivers in Japan. Int J Geriatr Psychiatry.

[40] Hayashi S, Terada S, Nagao S, Ikeda C, Shindo A, Oshima E (2013). Burden of caregivers for patients with mild cognitive impairment in Japan. Int Psychogeriatr.

[41] Yamashita M, Amagai M (2008). Family caregiving in dementia in Japan. Appl Nurs Res.

[42] Kamiya M, Sakurai T, Ogama N, Maki Y, Toba K (2014). Factors associated with increased caregivers’ burden in several cognitive stages of Alzheimer’s disease. Geriatr Gerontol Int.

[43] Hirono N, Tsukamoto N, Inoue M, Moriwaki Y, Mori E (2002). Predictors of long-term institutionalization in patients with Alzheimer’s disease: role of caregiver burden. No To Shinkei.

[44] Hosaka T, Sugiyama Y (1999). A structured intervention for family caregivers of dementia patients: a pilot study. Tokai J Exp Clin Med.

[45] Hashimoto M, Yatabe Y, Kaneda K, Honda K, Ikeda M (2009). Impact of donepezil hydrochloride on the care burden of family caregivers of patients with Alzheimer’s disease. Psychogeriatrics.

[46] Hishikawa N, Niwa H, Abe K (2014). Clinical effectiveness and caregiver burden of Galantamine. Prog Med.

[47] Kuroda A, Hokonohara D, Kurono A, Shinmon H, Yoshimuta N, Nagatomo I (2013). Effectiveness of rivastigmine from the point of caregiver for AD patients. J New Remedies Clin.

[48] Muramatsu N, Akiyama H (2011). Japan: super-aging society preparing for the future. Gerontologist.

[49] Takura T, Ushida T, Kanchiku T, Ebata N, Fujii K, DiBonaventura M (2015). The societal burden of chronic pain in Japan: an internet survey. J Orthop Sci.

[50] Vietri J, Otsubo T, Montgomery W, Tsuji T, Harada E (2015). The incremental burden of pain in patients with depression: results of a Japanese survey. BMC Psychiatry.

[51] Asami Y, Goren A, Okumura Y (2015). Work productivity loss with depression, diagnosed and undiagnosed, among workers in an Internet-based survey conducted in Japan. J Occup Environ Med.

[52] Charlson ME, Pompei P, Ales KL, MacKenzie CR (1987). A new method of classifying prognostic comorbidity in longitudinal studies: development and validation. J Chronic Dis.

[53] Quan H, Li B, Couris CM, Fushimi K, Graham P, Hider P (2011). Updating and validating the Charlson comorbidity index and score for risk adjustment in hospital discharge abstracts using data from 6 countries. Am J Epidemiol.

[54] Kroenke K, Spitzer RL, Williams JB (2001). The PHQ-9: validity of a brief depression severity measure. J Gen Intern Med.

[55] Muramatsu K, Miyaoka H, Kamijima K, Muramatsu Y, Yoshida M, Otsubo T (2007). The patient health questionnaire, Japanese version: validity according to the mini-international neuropsychiatric interview-plus. Psychol Rep.

[56] Manea L, Gilbody S, McMillan D (2012). Optimal cut-off score for diagnosing depression with the Patient Health Questionnaire (PHQ-9): a meta-analysis. Cmaj.

[57] Reilly MC, Zbrozek AS, Dukes EM (1993). The validity and reproducibility of a work productivity and activity impairment instrument. Pharmacoeconomics.

[58] Okuda M, Crawford B, Juniper E, Leahy MJ (2003). Preparation of Japanese versions of rinoconjunctivitis quality of life questionnaire (RQLQ) and work productivity and activity impairment questionnaire (WPAI-AS). Arerugi.

[59] Maruish ME (2011). User’s manual for the SF-36v2 Health Survey.

[60] Brazier J, Roberts J, Deverill M (2002). The estimation of a preference-based measure of health from the SF-36. J Health Econ.

[61] Walters SJ, Brazier JE (2005). Comparison of the minimally important difference for two health state utility measures: EQ-5D and SF-6D. Qual Life Res.

[62] Ferrara M, Langiano E, Di Brango T, De Vito E, Di Cioccio L, Bauco C (2008). Prevalence of stress, anxiety and depression in with Alzheimer caregivers. Health Qual Life Outcomes.

[63] Garcia-Alberca JM, Lara JP, Berthier ML (2011). Anxiety and depression in caregivers are associated with patient and caregiver characteristics in Alzheimer’s disease. Int J Psychiatry Med.

[64] Mahoney R, Regan C, Katona C, Livingston G (2005). Anxiety and depression in family caregivers of people with Alzheimer disease: the LASER-AD study. Am J Geriatr Psychiatry.

[65] Goren A, Gilloteau I, Lees M, DiBonaventura MD (2014). Quantifying the burden of informal caregiving for patients with cancer in Europe. Support Care Cancer.

[66] Gupta S, Goren A, Phillips AL, Stewart M (2012). Self-reported burden among caregivers of patients with multiple sclerosis. Int J MS Care.

[67] Gupta S, Isherwood G, Jones K, Van Impe K (2015). Assessing health status in informal schizophrenia caregivers compared with health status in non-caregivers and caregivers of other conditions. BMC Psychiatry.

